# Supplementary material for: Australian General Practitioners’ Current Knowledge, Understanding, and Feelings Regarding Breast Density Information and Notification: A Cross-Sectional Study
Source: Int J Environ Res Public Health. 2022 Jul 25;19(15):9029. doi: 10.3390/ijerph19159029 (PMC9332418; doi:10.3390/ijerph19159029)
Supplement: Supplementary file 1 [file ijerph-19-09029-s001.zip › ijerph-1797037-supplementary.pdf]

Australian primary care clinicians' current knowledge, understanding and feelings around breast density information and notification

SURVEY FOR PRIMARY CARE CLINICIANS (GPs)**:**

Survey Landing Page

Thank you for your interest in our study about breast density.

Please take the time to read through the Participant Information Sheet and Consent Form using the links below.

[Attach Participant Information Statement and Consent Form]

Being in this study is completely voluntary and you do not have to take part.

If you are interested in taking part in this study, you will be asked to consent to take part by ticking the 'yes' box at the beginning of the questionnaire. By giving your consent to take part in this study you are telling us that you:

- ✓ Understand what you have read in the Participant Information Statement and Consent Form.
- ✓ Agree to take part in the research study as outlined in Participant Information Statement and Consent Form.
- ✓ Agree to the use of your personal information as described.

When you have consented, you will fill out a questionnaire that asks some questions, such as:

- Clinician characteristics
- Breast density knowledge, prior experience and practices
- Attitudes and views towards notification
- Future information provision and training

You can withdraw your responses any time before you have submitted the questionnaire. Once you have submitted it, your responses cannot be withdrawn because they are anonymous.

If you would like to know more at any stage during the study, please feel free to contact Dr Hankiz Dolan (hankiz.dolan@sydney.edu.au) or Dr Brooke Nickel (brooke.nickel@sydney.edu.au)

The ethical aspects of this study have been approved by the human research ethics committee of the University of Sydney (Telephone: +61 2 8627 8176; Email: human.ethics@sydney.edu.au)

CONSENT
Do you consent to take part in this study as described in the Participant Information Statement and Consent Form?

a. Yes
b. No [Terminate the survey]

End of survey message: Thanks again for your interest in taking part in our study. Unfortunately, you do not meet our eligibility criteria for participating.

Section 1: Clinician characteristics

1. What type of practice, hospital or clinic do you work in?
   a. Public
   b. Private
   c. Both
   d. Other [Please specify – free text]

2. Is your primary practice a:
    a. Solo practice
    b. Group practice
    c. Hospital clinic
    d. Other

3. What is the postcode for the location of your primary practice?
    [free text]

4. What year did you complete your primary medical degree?
    [free text]

5. In which country did you complete your primary medical degree?
    [Dropdown menu]

    [If not in Australia]

    6. How many years have you held registration in Australia?
        ______years

7. How long have you been working in general practice in Australia?
    _______years

8. Do you have a special interest area of practice?
    a. No
    b. Yes [Please specify-free text]

9. How many hours on average per week do you currently work clinically?
    a. <10
    b. <10-19
    c. <20-29
    d. <30-39
    e. <40-49
    f. <50-59
    g. >60

10. Approximately, how many patients do you see each week?
    a. <50
    b. 50-99
    c. 100+

11. Approximately, what proportion of your practice would you say are women over the age of 40?
    a. <25%
    b. 25%-49%
    c. 50%-74%
    d. 75%-100%

12. Which of the following best describes your current gender identity?
    a. Male
    b. Female
    c. Non-binary/gender fluid
    d. Different identity
    e. Prefer not to say

Section 2: Knowledge

13. Prior to taking this survey, have you heard or read anything about the term ‘breast density’?
    a. Yes

b. No

[If a]

14. How did you hear or read about it? Please tick as many options as applicable.
    a. Medical school education
    b. Clinical work experiences
    c. Reading mammogram reports
    d. Attending professional and academic conferences
    e. Attending talks or seminars
    f. Reading journal articles or other academic/professional reading materials
    g. Talking to colleagues or other clinicians
    h. Personal experiences or experiences of close family or relatives
    i. Other [free text]

15. How well do you feel you understand breast density as a clinical issue at the moment? Using the following scale, please indicate the extent to which you understand breast density as a clinical issue.

No understanding		Average-level of understanding		High-level of understanding
1	2	3	4	5

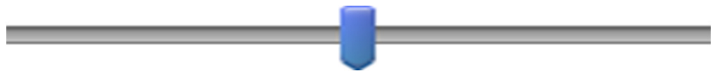

16. What is your understanding about how breast density is measured? Please tick as many options as applicable.
    a. Physical examination of the breasts
    b. Mammographic imaging
    c. Ultrasound imaging
    d. MRI imaging
    e. Digital breast tomosynthesis (3D mammogram)
    f. Other [please specify]
    g. Don't know

17. Have you heard of, or are you familiar with the Breast Imaging Reporting and Data System (BIRADS) system on classifying and reporting of breast density?
    a. Yes
    b. No

18. Having dense breasts can increase the risk of developing breast cancer.
    a. True
    b. False
    c. Don't know

19. If a woman has dense breasts, what impact does this have on the ability of a mammogram to correctly detect cancer?
    a. Dense breasts make it easier to see cancer on a mammogram
    b. Dense breasts do not impact the ability to see cancer on a mammogram
    c. Dense breasts make it more difficult to see cancer on a mammogram
    d. Don't know

20. Does a woman's breast density change with age?
    a. No, it does not change with age
    b. Usually increases with age
    c. Usually decreases with age
    d. Don't know

21. Prior to this survey, were you familiar with the concept of "supplemental screening" in the discussion of breast density? (i.e. offering patients with dense breasts a supplemental screening exam in addition to mammography in order to exclude breast cancer)
    a. Yes
    b. No

    [if a]
    22. Evidence suggests the following supplemental screening tests may be warranted for women with dense breasts (check all that apply):
        a. Ultrasound
        b. MRI
        c. Tomosynthesis (3D mammography)
        d. Genetic testing
        e. None of the above
        f. Don't know

Section 3: Prior experiences and practices

23. How often does a discussion about breast density typically occur in your own clinical practice?
    a. Every day
    b. About once a week
    c. About once a month
    d. About once a year
    e. Never. I have never discussed breast density with a patient

    [If e]
    24. Discussion about breast density never came up because:
        a. It was not reported on the mammogram reports
        b. Patients did not ask about it
        c. I do not have adequate knowledge about breast density and its implications
        d. I have not come across the idea of breast density as an issue to discuss with patients
        e. Other [Please specify-free text]

    [If a, b, c, d]
    25. How comfortable are you answering patients' questions about breast density?
        a. Very comfortable
        b. Somewhat comfortable
        c. Not comfortable
        d. I am not asked

    26. Which best describes your approach about supplemental screening exam?
        a. Every patient with dense breasts is offered a supplemental screening exam.
        b. Only certain dense breast patients are offered supplemental screening, based on unique patient/risk factors.
        c. Only some dense breast patients are offered supplemental screening, based on recommendation in the mammogram report
        d. Dense breast patients are not offered supplemental screening exams.

        [If a and b]
        27. Which supplemental screening modality do you most commonly suggest to your patients with dense breasts?
            a. Digital Breast Tomosynthesis (DBT)
            b. Breast Ultrasound
            c. Magnetic Resonance Imaging (MRI)
            d. None

28. When suggesting a particular supplemental imaging study, which one of the following most heavily influences your decision?
    a. Ease of obtaining exam
    b. Patient preference
    c. Patient's overall breast cancer risk
    d. Costs to patient
    e. I only have one exam to offer.
    f. Other (please specify)

29. How well equipped do you think primary care clinicians in your state are to discuss breast density with women at this time?

Not equipped at all		Fairly equipped		Highly equipped
1	2	3	4	5

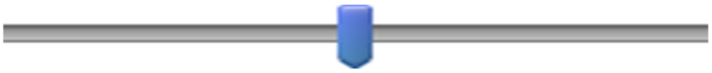

30. What do you think are/would be the key challenges in discussing breast density with patients? Please tick all that apply.
    a. Clinician's own lack of knowledge
    b. Patient's low health literacy
    c. Patient's low socio-economic background
    d. Lack of communication resources
    e. Lack of time
    f. No challenges
    g. Other [Please specify-free text]

Section 4: Attitudes and views towards notification

Breasts are considered dense if they have relatively high amount of fibrous and glandular tissue and relatively less amount of fatty tissue. The standard method of determining breast density level is by taking an x-ray/mammogram. Breast density cannot be determined by the physical examination of the breasts. There are four levels of breast density according to the BI-RADS (Breast Imaging Reporting and Database System): almost entirely fatty; scattered areas of fibroglandular density; heterogeneously dense; and extremely dense. The breasts usually get less dense as women get older.

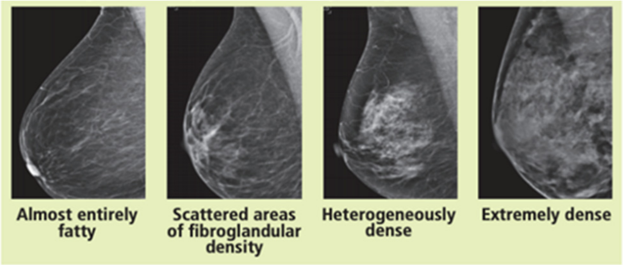

Source: American College of Radiology https://www.acr.org/-/media/ACR/Files/Breast-Imaging-Resources/Breast-Density-bro_ACR_SBI.pdf

Having dense breasts can increase women's risk of developing breast cancer. Dense breast tissue might make it harder to spot the lumps/tumours on the mammogram image and therefore, increases the chances of a breast cancer being missed on mammography.

It is common for women to have dense breasts. According to data from other countries, nearly half of the women over the age of 40 have dense breasts. Just like age and family history, breast density is not modifiable. Supplemental tests, such as breast ultrasound or MRI, or more frequent mammograms, might be able to improve the detection of cancer in women with dense breasts. However, supplemental tests increase women's chances of receiving false-positive results, being overdiagnosed and overtreated. There is uncertainty in whether supplemental

screening improves women's overall health outcomes. Mammogram remains the only screening method that is shown to reduce breast cancer deaths at a population level.

In the United States (US), the federal law mandates that screening services inform women if they have dense breasts. We don't have any legislation like this in Australia, and Breast Screen Australia does not routinely measure or report density, based on the best available evidence.

However, in Western Australia, BreastScreen WA has been notifying women of their density for years, and some imaging services (for example, radiology practices and private breast clinics) provide density information. A number of Australian consumer groups have been running campaigns to inform women about density and push for notification. BreastScreen Australia continues to consider whether reporting density is appropriate as the evidence base develops.

31. Do you believe that Australia should make it mandatory for publicly funded screening services to report breast density if women have dense breasts?
    a. Yes
    b. No [skip Q40]
    c. Don't know

Please read each of the following statements about breast density and its potential notification. Please choose the response that best describes how true each statement is for you.

	Strongly disagree	Somewhat disagree	Neither agree nor disagree	Agree	Strongly agree
32. I believe notifying women of their breast density will promote informed decision-making.	○	○	○	○	○
33. I am in favour of a policy to inform women of their breast density as part of their mammogram results.	○	○	○	○	○
34. Counselling women about breast density is primarily my responsibility.	○	○	○	○	○
35. Notifying women about their breast density may cause undue anxiety to them.	○	○	○	○	○
36. I believe women have the right to know about their breast density	○	○	○	○	○

If Australia were to adopt routine and widespread notification of breast density after each mammogram, how would this policy affect your clinical practice? Please read each of the following statements. Please choose the response that best describes how true each statement is for you.

	Strongly disagree	Somewhat disagree	Neither agree nor disagree	Agree	Strongly agree
37. This could affect my clinical practice.	○	○	○	○	○
38. I would feel prepared to respond to requests from patients who are notified that they have dense breasts.	○	○	○	○	○
39. I would need training in how to respond to requests from patients who are notified that they have dense breasts.	○	○	○	○	○

40. The breast density notification mandate may include several specific components. Which of the following statements do you think should be included in the letters provided to women? (check all that apply)

a. The mammogram showed dense breast tissue
b. The mammogram showed this particular degree or category of density
c. Dense breast tissue increases the risk of breast cancer
d. Dense breast tissue is common
e. Dense breast tissue can make it more difficult to find cancer on a mammogram
f. Additional screening may be advisable
g. Pros and cons of additional screening
h. MRI or ultrasound is the best means to find potential cancers in dense breasts
i. The patient should discuss results with their primary care physician or the referring physician
j. The patient has a right to discuss results with a radiologist

Future Information provision and training

41. Do you feel that you need or want more education about the issue of breast density and supplemental breast screening?
a. Yes
b. No

42. Which of the following would be most useful to alleviate confusion or improve the process of counselling patients regarding breast density and supplemental screening? (You may choose more than one answer.)

a. Synopsis of up-to-date scientific evidence
b. Interdisciplinary discussions with radiology department
c. Informational pamphlets/documents for patients
d. Conferences or workshops
e. Professional college educational programs like webinars or online CPD (Continuing Professional Development) courses
f. Nothing is needed; knowledge and current practices are appropriate
g. Other (please specify)

43. Do you have anything else to add on the topic of breast density or its potential widespread notification?
[free text] ______________________________

Feedback

44. Do you wish to receive feedback about the overall results of the study?
    a. Yes
    b. No

    (*if select 'yes' otherwise skip to end of survey)*

45. Please indicate your preferred form of feedback and address. Please note your personal information will be stored securely and kept separate from your survey responses, which will be stored anonymously.
    a. Postal________________________________

    b. Email_________________________________

46. If you wish to be entered into the prize draw for the chance to win one of five $100 vouchers, please provide your email address. Please note your personal information will be stored securely and kept separate from your survey responses, which will be stored anonymously.

    ☐ Email________________________________
